# Factors associated with undiagnosed hypertension among Tongan adults: a cross-sectional study

**DOI:** 10.1186/s41182-023-00570-4

**Published:** 2024-01-02

**Authors:** Seini Siahi Talanoafoou Fifita, Daisuke Nonaka, Mele Tilema Cama, Mele Inu Filise

**Affiliations:** 1 Graduate School of Health Sciences, University of the Ryukyus, Okinawa, Japan; 2 Faculty of Nursing and Health Sciences, Tonga National University, Nuku’alofa, Tonga; 3 Ministry of Health, Nuku’alofa, Tonga

**Keywords:** Undiagnosed hypertension, Prevalence, Risk factor, Tonga, Community-based survey

## Abstract

**Background:**

Hypertension is responsible for many premature deaths worldwide. However, many individuals with hypertension remain undiagnosed. Tonga is one of the countries that has had a steep increase in hypertension, thus undiagnosed hypertension could also be increasing. Purpose of this study was to assess the prevalence and factors associated with undiagnosed hypertension among Tongan adults.

**Methods:**

This cross-sectional study used data collected from conveniently sampled 473 participants using electronic questionnaire and digital sphygmomanometer through household visits between February and March 2023. Inclusion criteria were age of 18–65 years, residence in the villages for at least six months, and not being pregnant. Fisher’s exact test and mixed-effect logistic regression were performed using the EZR software to assess the association between undiagnosed hypertension and predictor variables.

**Results:**

The prevalence of undiagnosed hypertension was 22.4% (106/473). Five variables that were significantly associated with undiagnosed hypertension in Fisher’s exact test were included in the multivariate logistic regression. Overall, only three variables remained significant. First, participants who never had their blood pressure measured had higher prevalence compared to those who had it checked recently (33.3% vs. 19.1%); odds ratio: 2.24). Secondly, participants who were not aware of the risk of developing hypertension were significantly more likely to have undiagnosed hypertension compared to those who were aware (27.9% vs. 16.7%; odds ratio: 1.81). Lastly, middle-aged participants (30–49 years) and older (50–65 years), were significantly more likely to have undiagnosed hypertension compared to those who were 18–29 years old (30.0% and 23.7% vs. 11.8%; odds ratio: 3.58 and 3.38 vs. 1.00).

**Conclusion:**

The prevalence of undiagnosed hypertension could be substantial among Tongan adults, implicating a need to address this issue by doing further research and review current public health work to address hypertension in Tonga. Undiagnosed hypertension was associated with having no experience of blood pressure measurement, lack of awareness about hypertension, and age. Tongan government should provide people with more opportunities to have their blood pressure measured and to improve their awareness.

**Supplementary Information:**

The online version contains supplementary material available at 10.1186/s41182-023-00570-4.

## Background

Hypertension is one of the major causes of premature death across the globe, and 1.28 billion adults have been diagnosed with high blood pressure [1]. According to the World Health Organization (WHO), one of the global targets for non-communicable diseases (NCDs) is to control and decrease the prevalence rate of hypertension by 33% compared to 2010 by the year 2030 [1, 2]. Two-thirds of people with hypertension live in low- and middle-income countries [3], and it is a major burden in terms of economic development, social relationships, and most importantly, lives. Additionally, about 46% of adults who have hypertension are not diagnosed globally [1].

Undiagnosed hypertension occurs when those who have hypertension have not been informed of the condition by a health professional [4–6]. It is a silent but deadly condition that can lead to serious health consequences, including heart disease, stroke, kidney disease, and cognitive impairment [1]. Despite the availability of effective treatments for hypertension, undiagnosed hypertension remains a major health concern due to its lack of symptoms and low awareness among the general public [4, 7]. Hence, countries across the globe need to develop locally contextualized strategies to assess, monitor, and regulate the prevalence of undiagnosed hypertension [1, 8]. According to the first-ever global review of studies on hypertension 2019, 41% of women and 51% of men with hypertension remained undiagnosed in 200 countries, and regions of Oceania and sub-Saharan Africa had the highest prevalence of undiagnosed hypertension [9].

Factors associated with undiagnosed hypertension in previous studies include lifestyle factors such as eating habits, physical activity, alcohol consumption, and smoking [4–8]. Additionally, access to health care and the availability of health insurance in the context of social economic status have also been linked to undiagnosed hypertension [10–13]. Some of the other associated factors include age, sex, educational level, and household income [4–8, 10]. Most studies on undiagnosed hypertension have been conducted in high-income countries like Croatia [4], the United Kingdom [5], the United States [8], and France [10], although some studies have been done in low-middle income countries, including Indonesia [6], Fiji [7], Ethiopia [11], Nepal [12], and Peru [13].

Tonga is one of the Pacific Islands that has had a steep increase in hypertension from 1999 to 2019 [2, 3, 9]. Tonga is an archipelago that is part of Polynesia and is composed of 179 islands. The total population is 100,180, of which 74% reside on the main island of Tongatapu [14]. Health care services are predominantly provided by the government with one national referral hospital, three community hospitals, 34 reproductive health clinics, and 14 health centers [14, 15]. On Tongatapu, there are seven health centers located in rural areas that provide health services for nearby villages [14]. They are staffed by a nurse practitioner, health officers, and nurses and have routine outreach clinics to cater to the needs of the people in rural communities [14–16].

Although research addressing NCDs including hypertension has been prioritized, it is still slow and lacks contextualized evidence to aid practice in Tonga [15]. According to a recent census report, 77.1% were obese and 37% had hypertension [17]. Health education and programs for prevention are mainly run through the Ministry of Health with a priority of addressing NCDs [14]. This includes work on policy, food security, health care access, and the availability of health workers to improve efforts toward NCDs [15, 16].

The Tonga STEPS Surveys revealed an increase in the prevalence of hypertension from 27.6% in 2014 to 37.1% in 2017 [17]. However, little is known about the prevalence of undiagnosed hypertension and its risk factors [3]. The purpose of this study was to explore the prevalence and factors associated with undiagnosed hypertension among Tongan adults. Findings from this study could help to inform the Ministry of Health of Tonga in addressing prevention of undiagnosed hypertension and the promotion of awareness programs.

## Methods

### Study area

This cross-sectional study was done with a household survey that was completed from February to March 2023. The catchment area of the Nukunuku Health Center was chosen because it is located inland far from the western coastal area that experienced damage from a tsunami in 2022. Therefore, we thought the catchment area of Nukunuku health center is least likely to be impacted by the tsunami compared to other catchment areas. Additionally, it is 11.5 km away from the main hospital [14]. It contains six villages with a total population of 4190 people and 940 households [17]. Operational staff includes one nurse practitioner, two NCD nurses, and two reproductive health nurses. Daily, the health center is open for outpatient consultations from nine in the morning until five in the evening. Common prescription medications are also available except for the NCD medications which are often available from the main hospital [14].

### Participants

Households were selected with the help of town officers in the villages and health care workers at the Nukunuku Health Center to ensure that the selection met the criteria for inclusion in the study. Upon visiting households, two participants from the same household were invited to join this study, who were: (1) adults aged 18–65 years old; (2) residing in the villages for at least six months; (3) not pregnant; and (4) freely consenting to join the study. From each of the six villages, 40 households were included, accounting for 480 participants that had consented. Among the 480 participants, 473 were retained in the final analysis after excluding 7 participants who turned out not to meet the inclusion criteria.

### Variables and measurements

The outcome variable for this study was undiagnosed hypertension as individuals with hypertension who have never been told by a health professional that they have hypertension. Study participants with undiagnosed hypertension were defined as those who answered “No” to the question, “Have you ever been told by a doctor, nurse, or other health workers that you have raised blood pressure?” and showed hypertension during the survey. The operational blood pressure reading was based on the WHO’s definition of blood pressure reading for hypertension: blood pressure reading of systolic blood pressure ≥ 140 mmHg and/or diastolic blood pressure ≥ 90 mmHg [1]. According to the WHO’s protocol [18–20], blood pressure was measured twice during the household visit with a duration of at least 5–10 min apart. The averages of both measurements were used in the current study. The measurements were taken using an Omron 7120 digital arm sphygmomanometer with cuff sizes of 22–32 cm and 32–42 cm (Kyoto, Japan). Prior to the survey, calibration of the digital sphygmomanometers was done at the health center when testing the electronic questionnaire with 10 volunteers who visited the health center.

A total of 21 predictor variables were chosen based on similar studies that assessed factors associated with undiagnosed hypertension [4–8, 10–12]. In the current study, the questionnaire addressed these 21 predictor variables, which were self-reported during the household visit interviews. The predictor variables included age, sex, educational level, main source of income, health insurance status, ever having blood pressure measured by a health care professional, knowing common signs of hypertension, experiencing common symptoms, having visited a health center, knowing whether a health check-up is done at a health center, and feelings of difficulty or ease with travelling to the health center. Variables also included whether someone close to the interviewee has hypertension, whether someone close to the interviewee has other NCDs combined as cue to action, knowing the causes of hypertension, risk of cardiovascular disease with hypertension, and awareness of the risks of developing hypertension. The last part of the predictor variables included physical activity, consumption of fruits, consumption of vegetables, salt consumption, smoking, and consumption of alcohol.

### Data collection

Data were collected by trained surveyors (registered nurses) using an iPad with the electronic household questionnaire. The questionnaire (Additional file 1) was developed using the Epi Info 7 software and the STEPS Survey questionnaire as validation tool and guidance for the current study. Once households were identified, explanation sheets and consent forms were given, and at least 2–3 days later, surveyors visited to conduct the survey.

### Sample size

The sample size was calculated using a formula for logistic regression, *n* > 10* m*, where *n* is the smaller number of subjects with or without the outcome, and *m* is the number of predictor variables [21]. As 15 predictor variables that would be potentially included in a multivariate model, *n* should be over 150. Assuming that the prevalence of undiagnosed hypertension would be 40% [4–8, 10–12], at least 375 participants were needed. Assuming that the participation rate would be 80%, based on the participation rate of 85% in the Tonga STEPS Survey 2017 [16], at least 469 eligible people should have been invited. The sample size was increased to 480 people to protect against uncertainties in the sample size estimations. Since there were no similar studies or pilot studies done in Tonga, it was difficult to estimate the sample size for the current study. Therefore, sample size was slightly increased to prevent type II error, and to protect against uncertainties in the sample size estimations. Among the uncertainties special attention was given to the assumed prevalence of undiagnosed hypertension as the prevalence of undiagnosed hypertension varies across settings [9].

### Statistical analyses

Descriptive analyses were done to summarize the number and proportion of participants for each category. Bivariate analyses were performed to assess the association between the outcome variable and predictor variables using Fisher’s exact test. Multivariate analyses were conducted using logistic regression.

Only the predictor variables that were statistically significant in Fisher’s exact test were included in the multivariate analysis. A *p-value* < 0.05 was considered statistically significant. In the multivariate analyses, multi-level modeling was used to account for the hierarchical structure of the data: individuals were nested within households, and households were nested within villages. All the analyses were performed using the EZR software version 1.60 [22].

### Ethical consideration

Ethical approval was obtained from the National Health Ethics and Research Committee in Tonga, (Approval number 20221212) and from the Ethics Committee of the University of the Ryukyus for Medical and Health Research Involving Human Subjects (Approval number 2063). Participants were given both an explanation and written consent form, which was obtained before the data collection. Individuals in the current study who had shown undiagnosed hypertension were advised and referred to the health center for follow-up and treatment.

## Results

### General characteristics of participants

Approximately half of the participants (50.9%) were females (Table 1). The median age was 39 (inter-quartile range: 26 to 51) years. The most common education level was secondary school (70.2%), followed by technical vocational educational training (19.0%). The main source of income was civil servants (40.6%), followed by fishing (20.3%), farming (14.6%), and handicrafts (12.9%). Only 12.1% of participants reported having health insurance, and the main reason for not having it was not being able to afford it, followed closely by a lack of information.

**Table 1:** Demographic and socio-economic characteristics of participants

Characteristics	*n* (*n* = 473)	%
Sex		
Male	232	49.1
Female	241	50.9
Age		
18–29 years	152	32.1
30–49 years	190	40.2
50–65 years	131	27.7
Median (inter-quartile range)	39 (26–51)	
Education level		
Primary school	15	3.2
Secondary school	332	70.2
Technical vocational educational training	90	19.0
University	36	7.6
Main source of income		
Civil servant	192	40.6
Fishing	96	20.3
Farming	69	14.6
Handicrafts	61	12.9
Remittances	44	9.3
Others	11	2.3
Health insurance status		
Insured	57	12.1
Uninsured	416	87.9
Reasons for not having health insurance		
Cannot afford it	174	41.8
Lack of information about health insurance	144	34.6
Not aware about the options	92	22.1
Others	6	1.4

### Blood pressure reading and health conditions

The majority had their blood pressure measured by health professionals at some point (88.6%), and 71.3% of them had measurements done in 2023 or  2022 (Table 2). These readings were mostly performed in the main hospital (50.4%) and church screening (20.8%), and only 15.3% of the readings were performed at Nukunuku Health Center. A previous diagnosis of hypertension was recorded for 23% of participants, and 67.8% had been diagnosed between 2023 and 2022. The place of diagnosis was mainly the main hospital (43.1%), and only 19.3% had been diagnosed at Nukunuku Health Center.

**Table 2:** Table showing blood pressure and health conditions of participants

Characteristics	*n* (*n* = 473)	%
Ever had blood pressure measured by a health worker?		
Yes	419	88.6
No	54	11.4
When was the last measurement taken?		
2023	165	39.3
2022	134	32.0
2021	41	9.8
2020	26	6.2
2019	25	6.0
2018–2010	15	3.6
Do not remember	13	3.1
Where was the last measurement taken?		
Main hospital	211	50.4
Church screening	87	20.8
Nukunuku Health Center	64	15.3
Private clinics	28	6.7
Others	18	4.2
Home visit by nurses	11	2.6
Ever been told by health worker that you have hypertension?		
Yes	109	23.0
No	364	77.0
When were you told that you have hypertension? *(n* = 109)		
2023	38	34.8
2022	36	33.0
2021	7	6.4
2020	9	8.3
2019	9	8.3
2018–2015	7	6.4
2014–2010	3	2.8
Where were you told that you have hypertension? (*n* = 109)		
Main hospital	47	43.1
Nukunuku Health Center	21	19.3
Church screening	19	17.4
Private clinic	9	8.3
Others	9	8.3
Home visit by nurses	4	3.7
Ever had any chronic diseases that were confirmed at healthcare facility?		
Yes	91	19.2
No	382	80.8
Currently taking medication or under treatment for any chronic diseases?		
Yes	94	19.9
No	379	80.1
What chronic diseases are you currently taking medication or are under treatment for?^a^		
Hypertension	50	49.5
Diabetes	31	30.7
Cardiac disease	3	3.0
Others	17	16.9
Are you aware of the common signs of raised blood pressure?		
Yes	133	28.1
No	340	71.9
Do you sometimes have early morning headache?		
Yes	96	20.3
No	377	79.7
Do you sometimes have nose bleeds?		
Yes	15	3.2
No	458	96.8
Do you sometimes have shortness of breaths?		
Y es	113	23.9
No	360	76.1
Is the symptom (headache, nose bleed, and/or shortness of breath) severe or mild?^b^		
Mild discomfort	112	62.6
Moderate	20	11.2
Severe	26	14.5
Don’t know	21	11.7
Results of blood pressure measurement during survey		
Mean systolic blood pressure (standard deviation)	131 (17)	
Mean diastolic blood pressure (standard deviation)	83 (12)	
Undiagnosed hypertension		
Undiagnosed hypertension Have never been told by health worker that you have hypertension but showed hypertension during survey	106	22.4
Diagnosed and undiagnosed normal Have never been told by health worker that you have hypertension and did not show hypertension during survey	258	54.5
Diagnosed with hypertension Have been told by health worker that you have hypertension but did or did not show hypertension during survey	109	23.0

^a^The number of responses is not equal to the total number of participants as seven participants were taking medication for two or more diseases

^b^The number of responses is not equal to the total number of participants as only 179 answered “yes” to the questions about common symptoms

Only 19.2% had any chronic diseases that were confirmed at a healthcare facility, and 49.5% of them were taking medications for hypertension followed by diabetes. Most of the participants (71.9%) were not aware of the common signs of hypertension. Blood pressure readings during the survey revealed that the mean systolic blood pressure was 131 mmHg, and the mean diastolic blood pressure was 83 mmHg. The prevalence of undiagnosed hypertension was 22.4% (95% confidence interval (CI) 18.7% to 26.4%).

### Perception on difficulty of visiting Nukunuku health center

Most participants (67.4%) had visited the Nukunuku Health Center at some point (Table 3), especially within the past two years (75.3%). The top reason for visiting the health center was sickness (44.2%), and only 12.2% visited it for a health check-up. Among those who had visited the health center, nearly half (43.8%) were not satisfied with the services provided there. The most common reason for not being satisfied was the long waiting time (54.3%).

**Table 3:** Table showing the use and perception of Nukunuku Health Center (nearest healthcare facility)

Characteristics	*n* (*n* = 473)	%
Have you visited the health center?		
Yes	319	67.4
No	154	32.6
When was the last time you visited the health center?		
2023	125	39.2
2022	115	36.1
2021	14	4.4
2020	14	4.4
2019 or before 2019	18	5.6
Do not remember	33	10.3
Main reason for the visit		
Sick	141	44.2
Taking someone there	98	30.7
Health check-up	39	12.2
Others	28	8.8
Vaccinations	13	4.1
How satisfied are you with the services provided at the health center?		
Not satisfied at all	11	3.4
Not satisfied very much	129	40.4
Neutral	7	2.2
Satisfied	140	44.0
Very satisfied	32	10.0
Reasons for “not satisfied very much” or “not satisfied at all”		
Waiting time too long	76	54.3
Quality of service provided	11	7.9
Opening time not convenient	25	17.8
Other reasons	28	20.0
Yes	45	9.5
No	420	88.8
Did not answer	8	1.7
Reasons for giving up visiting the health center		
Quality of service provided	10	22.2
Lack of empathy and poor attitude	17	37.8
Others	18	40
Do you know if you can have a health check-up at the health center?		
Yes	261	55.2
No	212	44.8
How do you feel about the difficulty or ease of visiting the health center?		
Very difficult	16	3.4
Difficult	73	15.4
Not difficult but not easy	346	73.2
Easy	24	5.1
No answer	14	2.9
Can you use reliable transport if you need to get to the health center?		
Yes	375	79.3
No	82	17.3
Don’t know	16	3.4

Nearly half of participants (44.8%) did not know whether one can have a health check-up at the health center. Regarding the perceived difficulty or ease of visiting the health center, most participants (73.2%) perceived that it was “not difficult but not easy.” For most participants (79.3%), transport was available to travel to the health center.

### Health check-up and knowledge on hypertension

Over half of the participants (53.5%) had someone close to them with hypertension, and 53.3% had someone close to them with other NCDs (Table 4). Approximately half of the participants (51.2%) answered that they know a cause of hypertension, and the most reported cause was the consumption of salty food, followed by a lack of physical activity. Furthermore, 58.6% knew that having hypertension puts them at risk of developing cardiovascular diseases, stroke, and diabetes. Nearly half of the participants (49.3%) were aware of the risks of developing hypertension.

**Table 4:** Table showing cues to the action of health check-ups, knowledge about and perception of hypertension

Characteristics	*n* (*n* = 473)	%
Has someone close to you had hypertension and therefore visits the health center sometimes?		
Yes	253	53.5
No	212	44.8
Don’t know	8	1.7
Is there someone close to you with other NCDs?^a^		
Yes	252	53.3
No	211	44.6
Don’t know	10	2.1
Do you know about the causes of hypertension?		
Yes	242	51.2
No	231	48.8
Based on your knowledge, give at least two examples of causes of hypertension (multiple answers allowed)		
Obesity	70	14.5
Alcohol	69	14.3
Lack of physical activity	101	20.9
Consuming too much salty foods	114	23.6
Genetics	77	15.9
Co-morbidities	52	10.7
Other	1	0.2
Do you know that having hypertension puts you at risk for cardiovascular diseases, stroke, and diabetes?		
Yes	277	58.6
No	168	35.5
Others	28	5.9
Could you tell me your body height and weight?		
Yes, both weight and height	117	24.7
Yes, but weight only	104	22.0
Yes, but height only	1	0.2
No, neither of them	248	52.4
Don’t remember	3	0.6
Are you aware of the risks of developing hypertension?		
Yes	233	49.3
No	219	46.3
Other	21	4.4
Do you do any of the following actions to prevent hypertension?^b^		
Reduce salt intake	305	64.5
Become more physically active	288	61.0
Reduce trans-fat intake	174	36.8
Live a socially happy life	158	33.4
Work/life balance	120	25.4
Try to lose weight	111	23.5
Reduce consumption of red meat	110	23.3
Others	6	1.2

^a^Other non-communicable diseases: diabetes, cardiovascular, cancers, mental health issues

^b^Participants were allowed to choose multiple answers

### Lifestyle behaviors

Approximately 62.5% of the participants were physically active for at least 3 days per week (Table 5), and 8.3% were physically active daily for at least 30 min. Regarding consumption, only 11.4% ate fruits daily, and only 11.4% consumed vegetables daily. Most participants (63.3%) always, often, or sometimes add salt to their food before eating, more than half (58.4%) smoked daily, and 22.4% consumed alcohol. Among alcohol consumers, there were very few habitual drinkers; about 40.6% consumed it a few times per week.

**Table 5:** Table showing lifestyle behaviors of participants

Characteristics	*n* (*n* = 473)	%
How many days in a week do you do physical activity for 30 min or more?		
0	49	10.4
1	58	12.3
2	71	15.0
3	103	21.8
4	25	5.3
5	62	13.1
6	66	14.0
7	39	8.3
In a week, how many days do you eat fruits?		
0	28	5.9
1	81	17.1
2	148	31.3
3	89	18.8
4	28	5.9
5	28	5.9
6	17	3.6
7	54	11.4
In a week, how many days do you eat vegetables?		
0	14	3.0
1	50	10.6
2	109	23.0
3	114	24.1
4	82	17.3
5	39	8.2
6	11	2.3
7	54	11.4
How often do you add salt or salty sauce to your food before eating?		
Always	121	25.6
Often	86	18.2
Sometimes	92	19.5
Rarely	102	21.6
Never	71	15.0
Do not know	1	0.2
Do you currently smoke tobacco daily?		
Yes	276	58.4
No	197	41.6
Do you drink any alcohol, including beer, wine, or spirits?		
Yes	106	22.4
No	367	77.6
If yes, how often do you drink alcohol?		
Few times a week	43	40.6
Only at social gatherings	41	38.7
Only on special occasions	16	15.1
Daily	1	0.9
Few times a month	2	1.9
Others	3	2.8

### Fisher’s exact test

Bivariate analyses using Fisher’s exact tests showed that five predictor variables had a statistically significant association (i.e., *p-*value less than 0.05) with undiagnosed hypertension (Table 6). Hence, variables that were statistically associated were age, not having blood pressure checked before, feelings of difficulty with accessing health care services, cue of action to change behavior (i.e., the existence of someone close to a participant who had hypertension or other NCD), and awareness of developing hypertension.

**Table 6:** Table showing bivariate analysis: Fisher’s exact test on association of undiagnosed hypertension with predictor variables

Characteristics	Undiagnosed hypertension (*n* = 106)		Others (*n* = 367)		*p*-value
	*n*	%	*n*	%	
Age					
18–29 years	18	11.8	134	88.2	< 0.001
30–49 years	57	30.0	133	70.0	
50–65 years	31	23.7	100	76.3	
Ever had blood pressure measured by health worker?					
Yes, 2022 or 2023	57	19.1	242	80.9	0.043
Yes, 2021 or before/but don’t remember the year	31	25.8	89	74.2	
No	18	33.3	36	66.7	
How do you feel about the difficulty or ease of visiting the health center?^a^					
Very difficult/difficult	12	13.5	77	86.5	0.032
Not difficult but not easy/easy	90	24.3	280	75.7	
• Is there someone close to you who had hypertension and therefore visits health center sometimes? • Is there someone close to you with other NCDs?					
Both “Yes” to the above questions	28	16.1	146	83.9	0.035
“Yes” to either question	39	25.2	116	74.8	
“Yes” to neither question	39	27.1	105	72.9	
Are you aware of the risks of developing hypertension?					
Yes	39	16.7	194	83.3	0.004
No/others	67	27.9	173	72.1	
Sex					
Male	53	22.8	179	77.2	0.826
Female	53	22.0	188	78.0	
Educational level					
Primary and Secondary schools	84	24.2	263	75.8	0.135
Technical vocational educational training and University	22	17.5	104	82.5	
Main source of income					
Civil servant	38	19.8	154	80.2	0.312
Non-government occupations	68	24.2	213	75.8	
Having health insurance					
Yes	14	24.6	43	75.4	0.735
No	92	22.1	324	77.9	
Are you aware of common signs of raised blood pressure?					
Yes	26	19.5	107	80.5	0.392
No	80	23.5	260	76.5	
Do you have one or more of the following symptoms: headache, nose bleeding, and shortness of breath?					
Yes	43	24.6	132	75.4	0.424
No	63	21.1	235	78.9	
Have you visited the health center?					
Yes, 2022 or 2023	59	24.6	181	75.4	0.528
Yes, 2021 or before/but don’t remember the year	15	19.2	63	80.8	
No	32	20.8	122	79.2	
Do you know if you can have a health check-up at the health center?					
Yes	53	20.3	208	79.7	0.225
No	53	25.0	159	75.0	
Do you know about the causes of hypertension?					
Yes	62	25.6	180	74.4	0.098
No	44	19.0	187	81.0	
Do you know that having hypertension puts you at risk for cardiovascular diseases, stroke, and diabetes?					
Yes	60	21.7	217	78.3	0.656
No/Others	46	23.5	150	76.5	
How many days in a week do you do physical activity for 30 min or more?					
≤ 1	25	23.4	82	76.6	0.123
2–4	36	18.1	163	81.9	
≥ 5	45	26.9	122	73.1	
In a week how many days do you eat fruits?					
≤ 1	26	23.9	83	76.1	0.440
2–4	54	20.4	211	79.6	
≥ 5	26	26.3	73	73.7	
In a week, how many days do you eat vegetables?					
≤ 2	44	25.4	129	74.6	0.483
3–4	40	20.4	156	79.6	
≥ 5	22	21.2	82	78.8	
How often do you add salt or salty sauce to your food before eating?^b^					
Always	51	24.6	156	75.4	0.581
Sometimes	18	19.6	74	80.4	
Rarely/never	37	21.4	136	78.6	
Do you currently smoke tobacco daily?					
Yes	55	19.7	224	80.3	0.094
No	51	26.3	143	73.7	
Do you drink any alcohol, beer, wine, or spirits?					
Yes	23	21.7	83	78.3	0.895
No	83	22.6	284	77.4	

^a^ “No answer” was excluded

^b^ “Do not know” was excluded

### Results of logistic regression

Multivariate mixed-effects logistic regression analysis showed that three factors were significantly associated with undiagnosed hypertension (Table 7). The first factor was not having blood pressure checked before with odds ratios (OR) as follows; (OR 2.24, 95% CI 1.04–4.77) in comparison to those who did have it checked in 2022 and 2023. The second factor was not being aware about developing hypertension (OR 1.81, 95% CI 1.08–3.03) in comparison to those who were aware. Last factor was ages of 30–49 years (OR:3.58, CI 1.85–6.95) and 50–65 years (OR 3.38, 95% CI 1.63–6.99), which were associated with higher chances of having undiagnosed hypertension compared to those aged 18–29 years.

**Table 7:** Logistic regression analysis of factors associated with undiagnosed hypertension

	Bivariate analysis		Multivariate analysis	
Variables	Odds ratio	95% CI^a^	Odds ratio	95% CI^a^
Ever had BP^b^ measured?				
Yes, in 2023 or 2022	1.00	Reference	1.00	Reference
Yes, in 2021 or before	1.48	0.90–2.44	1.11	0.63–1.97
No	2.12	1.12–4.01	2.24	1.04–4.77
Awareness of developing hypertension				
Yes	1.00	Reference	1.00	Reference
No/others	1.93	1.23–3.01	1.81	1.08–3.03
Age (years)				
18–29	1.00	Reference	1.00	Reference
30–49	3.19	1.78–5.71	3.58	1.85–6.95
50–65	2.31	1.22–4.36	3.38	1.63–6.99
Feelings on difficulty of visiting health center^c^				
Very difficult/difficult	1.00	Reference	1.00	Reference
Not difficult but not easy/easy	2.06	1.07–3.96	1.96	0.93–4.11
Has someone close to you had hypertension and visits the center sometimes? Does someone close to you have another NCD^d^?				
Both yes to the above questions	1.00	Reference	1.00	Reference
Yes to either of the above questions	1.75	1.02–3.02	1.60	0.85–3.01
Yes to neither question	1.94	1.12–3.34	1.77	0.92–3.43

^a^CI: confidence interval

^**b**^BP: blood pressure

^**c**^“No” answers were excluded

^**d**^NCD: non-communicable disease

## Discussion

The results showed that 22.4% (106/473) of the study population had undiagnosed hypertension. In a global review of hypertension, the denominator used was only the people who had both been diagnosed before and were undiagnosed [9]. When applying this to the current study, the percentage of undiagnosed hypertension is 49.3% (106/215). The current results show that undiagnosed hypertension is a significant public health problem that needs to be addressed by the Tongan government. According to the results, undiagnosed hypertension was significantly associated with three variables: no experience of blood pressure measurement, a lack of awareness of developing hypertension, and age.

Participants who never had their blood pressure measured before were significantly more likely to show undiagnosed hypertension compared to those who had it checked recently (33.3% vs. 19.1%). This finding is in line with the statement of the International Society of Hypertension that every adult should know their blood pressure as a starting point to address hypertension [23]. In response to this, an international screening campaign for hypertension was launched from 2017 to 2019, which found that 32.0% had never had their blood pressure measured [24].

Additionally, studies in Fiji [7], France [10], the United States [8], and China [25] reported that individuals who had regular visits to their doctors and health centers had more opportunities for regular health check-ups. One of the other documented reasons for this is that hypertension is silent and asymptomatic, so individuals usually do not see the need to regularly visit their healthcare facilities [6, 7, 11]. It is startling that even though simple inexpensive regular health check-ups can contribute to early detection, prevention, and management of hypertension, it is not being utilized well in many low- and middle-income countries. As suggested in many reports by the WHO, each country needs to contextualize its response plans and strategies to address hypertension. Tonga is a small island country with a population of 100,180 people and faces high mortality from coronary heart diseases. Early detection, prevention, and management of hypertension can contribute well to the improvement of cardiovascular diseases.

The second factor was a lack of awareness about developing hypertension. This finding is in line with a review examining hypertension in low- and middle-income countries, which stated that the first barrier to addressing hypertension is poor awareness [26]. In the current study, of the participants who had visited the health center, 78.7% answered that they had not seen or could not remember seeing, hearing, or receiving health promotion activities for hypertension while visiting the center. In previous evaluation studies in other countries on the effectiveness of awareness campaigns, the campaigns had positive impacts on early detection, prevention, and treatment of hypertension [25–29]. It is also evident from previous studies and reviews in low- or middle-income countries have limitations in terms of both human and financial resources to combat hypertension.

The last associated factor for undiagnosed hypertension in the current study was greater age. One of the reasons for older adults having undiagnosed hypertension is that as one ages, arterial stiffness contributes to increases in blood pressure [3]. In studies in France, Ethiopia, and Fiji, reasons given for middle-aged adults (30–50 years) having undiagnosed is the assumption that they are healthy, so they do not go for regular check-ups [7, 10, 11, 23–25]. The trend of undiagnosed hypertensive Tongan adults from the current study is consistent with this finding of adults aged 30–49 years showing higher prevalence of hypertension.

Only 12.1% of the participants had health insurance (Table 1). The result is consistent with that of the Tonga STEPS Survey 2017 that reported that only 11.3% of the survey respondents had health insurance [16]. Currently, in Tonga, health care is free for all citizens [15] and could be the reason why Tongan people do not include health insurance in their budget. In previous studies, having health insurance had positive impacts on health outcomes and the frequency of visits to health care providers [30–35]. Thus, it could be a contributing factor for Tongan adults not having regular health check-ups because of a lack of health insurance scheme, but further research and exploration are needed. According to the present study, the most common reason for not having health insurance was affordability. Therefore, the government should consider providing affordable health insurance to promote regular health check-ups.

In the current study, most diagnoses of hypertension were made at the main hospital (43.1%), followed by church screening (20.8%) and health center (15.3%) (Table 2). It is natural for screening to be done in hospital and health center settings, but it is evident from the current study that church screening can contribute to early diagnosis of hypertension. Although screenings are done, more than half of participants (52.4%) were not aware of their own blood pressure readings and about the next steps to take.

This study had two main limitations. First, this study used a non-probability sampling method, so sampling bias is a concern. However, the impact of the bias on the study findings would be small as 240 out of a total of 940 households were covered by the study. Furthermore, no household rejected the invitation to study. Second, this study was limited to one health-center zone on the main island, so the applicability of the findings to wider areas of Tonga would be a concern. However, we believe that the findings of this study can be applied to other health-center zones on the main island as socio-economic situations are not greatly different between zones.

### Recommendations

Three main recommendations from this study, firstly is the promotion and advocation of regular health check-ups. As shown in studies in other countries like Japan [31], China [36], and Vietnam [37], people who have had regular health check-ups have earlier detection and better management of hypertension. Secondly, government and ministry of health Tonga should empower and streamline regular health check-up [25–27] process and sharing of information is recommended to enhance continuity of health care [28, 29, 33–36]. Lastly is to encourage young and older adults to participate and raise awareness on hypertension since they are the age group mostly associated in this current study with undiagnosed hypertension.

Considering the limitations of the current study, we propose further research using a randomly selected population sample from the wider areas of Tonga, so that we confirm the findings of the current study.

## Conclusion

The prevalence of undiagnosed hypertension is substantial among the Tongan adults examined in this study, indicating a need to address this issue. Findings from this study implicate a need to revisit public health measures on prevention, early detection, and management of hypertension. For instance, having more consistent health check-up opportunities and awareness campaigns using the young and older adults as target groups. Undiagnosed hypertension was significantly associated with not having blood pressure measured, a lack of awareness about hypertension and age groups of middle-aged and older adults. The Tongan government should provide people with more opportunities to have their blood pressure measured and to improve awareness. This could contribute to the fight to control the burden of premature deaths in the country.

### Supplementary Information

**Additional file 1. **Questionnaire for Undiagnosed Hypertension Amongst Tonga adults: A Cross-Sectional Study Feb–Mar 2023.

## Data Availability

The datasets generated or analyzed in the current study are available from the corresponding author upon a reasonable request.
