# Supplementary material for: Factors associated with undiagnosed hypertension among Tongan adults: a cross-sectional study
Source: Trop Med Health. 2024 Jan 2;52:4. doi: 10.1186/s41182-023-00570-4 (PMC10759494; doi:10.1186/s41182-023-00570-4)
Supplement: Supplementary file 1 — Additional file 1. Questionnaire for Undiagnosed Hypertension Amongst Tonga adults: A Cross-Sectional Study Feb–Mar 2023. [file 41182_2023_570_MOESM1_ESM.docx]

***Questionnaire for Undiagnosed Hypertension amongst Tongan adults: A Cross-Sectional Study Feb-Mar 2023.***

***SECTION 1: PROFILE DETAILS.***

**Participant code:**

* First digit: Village of Participant, Digit 2-3: household number in the village, digit 4-5 participant number.

**Age:**

**Gender:**

1. Female

2. Male

1. Other

**1. What is your highest educational attainment?**

1.Primary

2.Secondary

3.TVET

4.University

5.Others (specify)

***SECTION 2: Health history.***

***Undiagnosed Hypertension****.*

**2.Have you ever had your blood pressure measured by a doctor, nurse or other health worker?**

1.Yes

2.No

*(If the participant answers yes then the branching question 2.1 should be answered, if one answers no then the iPad should take you to the next question).*

**2.1 Please tell me when you had your last measurement taken?**

1. 2023
2. 2022
3. 2021
4. 2020
5. 2019
6. 2018-2015
7. 2014-2010
8. cannot remember

**2.2 Where was the latest measurement taken?**

1. Nukunuku Health Center
2. Other Health Center

3. Main hospital

4. Private Clinic

5. Overseas

6. Home visit by nurse

7.Church Screening

8. Other (specific in the next column

**3. Have you ever been told by a doctor, nurse or other health worker that you have a raised blood pressure or hypertension?**

1.Yes 2.No

*(If the participant answers yes then the next question 3.1 should be answered, if the answer is no pressing tab on the iPad should take you the next question).*

- 1. **When were you told that you had raised blood pressure?**

1.2023

2. 2022

3. 2021

4.2020

5. 2019

6. 2018-2015

7. 2014-2010

8. Cannot remember

- 1. **Place where you were told that you had a raised blood pressure**?

1. Nukunuku Health Center

2. Other health Center

3. Main hospital

4. Private clinic

5. Overseas

6. Nurses home visit

7. Church Screening

8. Others

- 1. Specific Textbox if others was chosen.
  2. **4.4 Blood pressure readings.**

1st Reading- Diastolic value: systolic value:

2nd Reading- Diastolic value: Systolic value:

Average Diastolic reading: Average Systolic reading:

**5. Have you had any chronic disease that was confirmed at a healthcare facility?**

1.Yes

2.No

3.Do not know

*(If participant answers yes and then the branching question 5.1 should be answered, if answering no then the tab should take you to the next question).*

- 1. **Are you currently taking medications or under treatment for any chronic disease (s)?**

1.Yes 2. No

**5.2. What chronic disease(s) are you currently taking medication or under treatment?**

*(Choose the chronic diseases that participant may have)*

1.Hypertension

2.Diabetes

3.Cancers (all types)

4.Cardiac diseases (all-types)

5.Respiratory problems

6.Mental Health Issues.

7. Trauma all types

8. Strokes

9. Others

5.3 Specific box if others is chosen then list answers here.

***PHYSICAL ACCESSIBLITY TO THE HEALTH CENTER.***

**6. How do you feel about the difficulty or easiness of visiting Nukunuku Health Center?**

1.Very difficult

2.Difficult

3.Not difficult but not easy

4.Easy

5.Very easy

**7. Can you use reliable transport so that if needed you get to the health center?**

1.Yes 2.No 3. Do not know

***SATISFACTION WITH THE HEALTH CENTER.***

**8. Have you visited or seek healthcare from Nukunuku Health Center in the past?**

1. Yes 2. No

*(If answer to question 8 is no then branching questions 8.1 and 8.2 is not answered)*

**8.1 How much are you satisfied with the services provided at the Nukunuku health center?**

1. Not satisfied at all

2. Not satisfied very much

3. Neutral

4. Satisfied

5. Very satisfied.

*(If answering with either 1 or 2 then branching question 8.2 should be answered and if other answers are given then tab should take you to the next question)*

**8.2 If you had answered that you are not satisfied very much or not satisfied at all please give reason.**

1.Waiting time too long.

2.Quality of service provided.

3.Opening time not convenient

4.Worried about privacy

5.Lacks empathy and poor attitude

6.Other reasons (specify)

8.3. Textbox for if the participant chose other reasons.

**9. Have you ever given up visiting the health center when you want to seek health care?**

1.Yes

2.No

3.Do not know

*(If answering yes to question 9 then branching question 9.1 should be answered, if other answers are provided then tab should take you to the next question)*

**9.1 If you had answered yes to question 9, please state the reason here.**

1.Waiting time too long.

2.Quality of service provided.

3.Opening time not convenient

4.Worried about privacy

5.Lacks empathy and poor attitude

6.Other reasons (does not meet my needs)

**10. Do you know if you can have a health check-up at Nukunuku Health Centre?**

1.yes

2.no

***HEALTH CARE VISITS.***

**11. When was the last time you had a visit to the health center?**

1.2023

2. 2022

3. 2021

4.2020

5. 2019

6. 2018-2015

7. 2014-2010.

8. Do not remember.

9. Have not visited.

**11.1 Main reason for visiting the health center.**

1.Sick

2.Health check-up

3.Taking someone there

4.Vaccinations

5. Seeking information

6. Others (specify)

**11.2 Specific Box for question 11.1 if others were chosen.**

**12. During your visit to the health Centre did you hear or see any information, promotion or activities about raised blood pressure?**

1.Yes

2.No

3. Do not know

***SEVERITY OF SYMPTOMS.***

**13. Are you aware of the common signs of raised blood pressure?**

1.Yes

2.No

**14. Do you sometimes have early morning headaches?**

1.Yes

2.No

**15. Do you sometimes have nose bleeds?**

1.Yes

2.No

**16. Do you often have shortness of breaths?**

1.Yes

2.No

*(If the participant had answered no to all of the questions from 13-16 then question 17 should be disabled than tab should take you to the next question).*

**17. If you had answered yes to the previous questions, do you think that it is severe or mild?**

1.Mild discomfort

2.Moderate

3.Severe

4.Do not know

**18. If you experience any of the symptoms where would you prefer to seek help?**

1. Private clinic

2.Preacher

3.Traditional healer

4. Main Hospital

5.Nukunuku HC

6.Another health Center.

***CUE TO ACTION.***

**19. Do you have someone close to you who have raised blood pressure and often visits the health center?**

1.Yes

2.No

3. Do not know

**20. Do you have someone close to you having other NCDs (diabetes, cardiovascular, cancers, mental health issues)?**

1. Yes

2. No

3. Do not know

*(In these questions someone close to you is defined as family, friends, relatives or from the community that the participant knows)*

***KNOWLEDGE ABOUT HYPERTENSION.***

**21. Do you know about the causes of raised blood pressure?**

1.Yes

2.No

*(If the participant answers yes to question 21 then the branching question 21.1 should be answered if not then tab should take you to the next question).*

**21.1 Based on your knowledge, give at least two examples of the causes of hypertension?**

*(You can choose at least 2 answers).*

1.obesity

1. alcohol
2. lack of physical activity
3. salty food consumption
4. Genetics
5. Other diseases (NCD)
6. other reasons (specify)

*(In question 21.1 although the answers are provided but please do not provide the answers or choices as you are testing to see their knowledge, give them time to think and provide the answer).*

21.2 (textbox for others should be available for you to input if participant chooses others)

**22. Do you know that having raised blood pressure puts you at risk for cardiovascular diseases, stroke and diabetes?**

1.Yes

2.No

3. Others (no clear answer given, do not understand the meaning of risks).

***AWARENESS ON THEIR HEALTH CONDITION***

**23. Could you tell me your body height and weight?**

**(Please enter the exact readings if known) or (not aware)**

**24. Are you aware of the risks to having a raised blood pressure?**

1.Yes

2.No

3.Others (no clear answer given, do not understand the meaning of risks).

**25. Do you do any of the following actions to prevent having raised blood pressure?**

**(Allowed to choose more than 1 answer)**

1.reduce salt intake

2. reduce consumption of red meat.

3. reduce trans-fat intake

4. become more physically active.

5. try to lose some weight

6. live a socially happy life.

7. avoiding stress

8. work life balance

9.others

*(Question 25, allow participants to choose more than 1)*

25.1 Specific textbox for question 25

***SECTION 3: Variables from other research.***

**HEALTH INSURANCE.**

**26. Do you have health insurance?**

1.Yes

2.No

3. Do not know

*(If the participant answers no than the branching question 26.1 should be asked if the other answers are given then tab should take you to the next question).*

**26.1. If you answered no, please state reason of not having health insurance:**

1. Cannot afford

2.Lack of information about health insurance

3.Not aware about the options

4. Other (Specify)

**PHYSICAL ACTIVITY.**

**27. How many days of the week do you do exercise for at least 30 minutes a day? Number of days.**

**FOOD CONSUMPTION.**

**Fruit & Vegetable Consumption.**

28. In a week how many days do you eat fruit? (Number of days)

29. In a week how many days do you eat vegetables? (Number of days).

**30. How often do you add salt or salty sauce such as soy sauce to your food right before you eat it or as you are eating it?**

1.Often

2. Rarely

3. Sometimes

4. Always

5. Never

6. Do not know

**31. How often is salt, salty seasoning or salty sauce added in cooking or preparing food in your household?**

1.Often

2. Rarely

3. Sometimes

4. Always

5. Never

6. Do not know

**SMOKING & ALCOHOL INTAKE.**

**Smoking.**

32. Do you currently smoke tobacco daily?

1.Yes

2.No

**Alcohol Consumption.**

**33. Do you drink any alcohol such as beer, wine, spirits or any other local alcohol?**

1.Yes

2.No

**33.1. If you answered yes to the previous question, how often do you drink alcohol?**

1. Daily
2. Few times a month
3. Only on special occasions
4. Few times a week
5. Only on social gatherings
6. Other (specify)

***SECTION 4: HOUSEHOLD INCOME & LIVING ARRANGEMENTS*.**

**34. In this household who is taking care of your finances?**

1. mother
2. father
3. son.
4. Daughter
5. 5.other (specify)

34.1 Specific Text box if others is chosen.

**35. How many people are living in this household?**

(Number of people here)

**36. How many of them are 18 and above years old**?

(Number of people)

**37. What is the main source of income for this household?**

1. Civil Servant
2. Remittances
3. Fishing
4. Farming
5. Handicrafts
6. 5.Other (specify)

*(For question 37 allow remind the participant that this is for the MAIN income only)*

**38. Approximately how much income (in TOP) is being earned per year for your household?** (Please type or enter the approximate amount)

*(Question 38 is asked to the participant if the*

*person who handles the finances is not present than they can be allowed to call them and ask for the answers if they do not know it, especially for income.*
